# Representation of autism in fictional media: A systematic review of media content and its impact on viewer knowledge and understanding of autism

**DOI:** 10.1177/13623613231155770

**Published:** 2023-02-19

**Authors:** Sandra C Jones, Chloe S Gordon, Simone Mizzi

**Affiliations:** Australian Catholic University, Australia

**Keywords:** attitudes, autism, fictional media, knowledge, representation

## Abstract

Media depictions of autism have the potential to have either positive or detrimental impacts on the lives of autistic people. This systematic review aimed to examine (1) the accuracy and authenticity of fictional media portrayals of autism (Part A) and (2) the impact of viewing such portrayals on knowledge about autism and attitudes towards autistic people (Part B). Seventeen articles met the inclusion criteria and were included in the review: 14 for Part A and 3 for Part B, comprising five experimental studies. The media reviewed in Part A covered a 30-year period (1988–2017). Several stereotypical and unhelpful portrayals surfaced. Positive portrayals of autism highlighted strengths and reflected nuance. Overall, participants in the fiction conditions in Part B were significantly more likely to attribute positive traits to an autistic person than those in the control conditions, with medium effect sizes ranging from 0.57 to 0.73. There were no improvements in knowledge. Given the small number of studies included in this review, further evidence is needed to understand the conditions under which fictional media may be effective. There is also a need to develop measures that accurately and respectfully measure autistic people’s experiences of community knowledge and attitudes.

**Lay abstract:**

The way autism is represented in fictional media can impact people’s views of autistic people. For example, representations may contribute to negative views of autistic people as being unusual or dangerous, or they may challenge stereotypes and instead highlight the strengths of autistic people. This work aimed to review previous research to understand how autistic people have been represented in fictional media (Part A). It also sought to understand whether viewing fictional portrayals of autism has an impact on people’s knowledge of autism and attitudes towards autistic people (Part B). Of 14 studies that were included in Part A, several unhelpful and stereotypical portrayals of autism emerged. Positive portrayals were those that highlighted the strengths of autistic people and reflected nuance. There is a need for greater diversity in representation of autism in fictional media. For example, not all autistic people are white heterosexual males. Across the five studies included in Part B, there were no improvements in people’s knowledge of autism after watching or reading a short segment from a fictional TV series or novel that depicts an autistic person. Although there was a significant improvement in people’s attitudes towards autistic people, these findings do not provide a complete picture given the short length of the media exposure and small number of studies. Future studies should investigate how multiple exposures to the representation of autistic people in both fictional and non-fictional sources can affect people’s understanding of autism. There is also a need to develop more accurate and respectful ways of measuring people’s knowledge of, and attitudes towards, autism.

The media has a significant and powerful influence on shaping societal beliefs and attitudes towards others (Baroutsis et al., 2021; Fontes & Pino-Juste, 2021). This influence is particularly strong when a person’s direct experience with the ‘other’ is limited (Baroutsis et al., 2021). Although most people have heard of Autism Spectrum Condition (hereafter termed ‘autism’), society still has a considerable way to go in understanding the needs of autistic people (Jones, Akram, et al., 2021) and accepting and including autistic people of all ages (Jones, Gordon, et al., 2021). In an Australian study with 1297 autistic people and parents and carers of autistic people, autistic adults reported feeling socially isolated and reluctant to leave their houses because of concern about how they will be treated. Parents and carers similarly reported negative reactions when in the community with their family (Jones, Gordon, et al., 2021).

Media depictions of autism have the potential to have either positive or detrimental impacts on the lives of autistic people. For example, they have potential to be used as educational tools to improve understanding of the strengths and challenges experienced by autistic people. However, their effectiveness as an educational tool is likely dependent upon the educator’s own knowledge and ability to guide the students to distinguish between fictional and actual cases of autism (Nordahl-Hansen, Tøndevold, & Fletcher-Watson, 2018). Media depictions may also reinforce negative stereotypes of autism, thereby leading to inaccurate views of autism and increasing stigma (Nordahl-Hansen, Øien, & Fletcher-Watson, 2018). A study reporting on the experiences of autistic higher education students found that stereotyping was one of the reasons students chose to not disclose their diagnosis to their peers (Van Hees et al., 2015). Negative stereotypes are also problematic as they can lead to negative behaviour towards others (Treweek et al., 2019), with stereotypes dehumanising autistic people and being used to justify prejudice and discrimination (Treweek et al., 2019).

A scoping review examined available literature published up until the end of 2019 on the portrayals of autism in media and the impact of such portrayals (Fontes & Pino-Juste, 2021). Across the 22 publications included in the review, 13 examined fictional examples of autism, while the remainder analysed newspapers, news broadcast and a videoclip. The articles were discussed under five categories: stereotypes, stigma, social attitudes, quality, and benefits and downsides. Several autistic stereotypes were identified, such as the intellectual genius and the dangerous and incontrollable. Stigma was identified across the media types, although was particularly present in newspapers and broadcast news. The impact of autism portrayals on social attitudes was explored in three instances, while the quality of portrayals was predominantly evaluated against the *Diagnostic and Statistical Manual of Mental Disorders, 5th Edition* (DSM-5) criteria. Opinions on the educational potential of media portrayals varied, with some publications emphasising that portrayals are unrepresentative and therefore unhelpful, while other publications highlighted potential benefits of the portrayals. This study provides a helpful starting point for understanding the body of literature reporting on media portrayals of autism. However, methodological limitations of the review, including the limited search strategy and lack of clear inclusion and exclusion criteria, make it difficult to draw strong conclusions.

Another review examined the state of current research on autism representation in film and television up until April 2020 (Dean & Nordahl-Hansen, 2021). More specifically, the review focused on the demographic attributes of the autistic characters discussed in the articles. Eighty-seven autistic characters were discussed across the 25 publications included in the review. The review found that although stereotypical representations were evident, there was growing diversity among representations, such as autistic characters from non-Caucasian backgrounds and Lesbian, Gay, Bisexual, Transgender, Queer, Intersex, Asexual+ identifying, although these minority groups were still underrepresented. There was also evidence of a movement away from historically common disability tropes such as the autistic savant.

The current study seeks to build upon previous reviews to examine the accuracy and authenticity of fictional media portrayals of autism across all media types (i.e. film, television, novels, picture books etc.). Including a range of fictional media, rather than only focusing on the traditional media platforms of film and television, was considered important given that educational contexts are required to expose students to a variety of text types (Strachan, 2014). Measuring the ‘accuracy’ of fictional portrayals has its limitations and pitfalls, in terms of both what is being assessed and how it is being measured. The process of diagnosing a (real) person with autism is complex and involves extended observation and interaction, a process that is not possible with a fictional portrayal. As with the problematic practice of retrospectively ‘diagnosing’ historical figures, the accuracy cannot be definitively determined in the absence of the living person (Muramoto, 2014; Palermo & Bogaerts, 2015). We also recognise that the medical model of autism, as epitomised in the DSM, is contentious in treating autism as a pathology rather a normal variation in the human condition as reflected in the social model of disability and the neurodiversity movement (Dyck & Russell, 2020; Leveto, 2018). While acknowledging these limitations, we feel that the issue of accuracy is relevant given the powerful impact of media on societal beliefs and attitudes; that is, a grossly inaccurate portrayal of autism would increase misperceptions among viewing audiences.

The study also seeks to extend previous reviews by including a second component that examines the impact of viewing fictional media portrayals of autism on knowledge about autism and attitudes towards autistic people. Findings are intended to inform the development of principles to guide the selection of fictional representations of autism in educational contexts. Given the isolation and discrimination faced by autistic people, a careful and informed selection of media texts may be one way in which knowledge and acceptance of autism can be improved.

## Method

This systematic review has been written in accordance with the 2020 Preferred Reporting Items for Systematic Review and Meta-Analysis statement (PRISMA) guidelines (Page et al., 2021). Prior to formal commencement of the study, a protocol was published on the Open Sciences Framework database (https://osf.io/bwdmr). The research questions that guided the review were the following:

**Research Question 1 (RQ1):** How accurate and/or authentic are fictional media portrayals of autism? (Study Part A)
**Research Question 2 (RQ2):** What is the impact of viewing fictional media portrayals of autism on knowledge about autism and attitudes towards autistic individuals? (Study Part B)

### Search strategy

A database search strategy was developed in consultation with a research librarian (see Table 1). Thirteen electronic databases (Scopus, ERIC, PsycINFO, CINAHL, Web of Science, MEDLINE Complete, JSTOR, Emerald Insight, PsycExtra, Project Muse, Humanities International Complete, Communication & Mass Media and Academic Search Complete) were searched from database inception to February 2021. A search of grey literature was also conducted through ProQuest Dissertations & Theses and Google databases (only the first 100 results) to reduce the impact of publication bias. This search was repeated in October 2021. The full search strategy of all databases can be seen in Supplementary File 1.

**Table 1.** Key concepts and terms used in search.

	Concept 1: Media	Concept 2: Autism	Concept 3: Representation and impact
**Key terms**	media TV television entertainment movie* film* fiction* "popular culture" drama	ASD autis* asperger*	knowledge attitude* influenc* perception stigma understand* awareness belief portray* stereotyp* depict* represent* presentation fram*

### Inclusion/exclusion criteria

To be included, articles were required to have been available in English and have a focus on fictional media representations of autism (fictional media portrayals of autistic characters) on media platforms, such as television shows, movies and novels. To be included in Part A, studies had to use a clear coding framework to measure the accuracy and/or authenticity of the portrayal of autism (a pre-established tool such as the DSM-5 criteria, or a tool purpose-built for the study). Accuracy is defined as containing ‘correct’ information about autism spectrum disorder (ASD), while authenticity is defined as the media text being ‘believable’ and a ‘true portrayal of ASD’ (Cardon & Kelley, 2016). To be included in Part B, studies needed to include a control group, and measure the impact of viewing the fictional media portrayal on knowledge of, or attitudes towards, autistic people, as measured through behavioural checklists, checklists of positive and negative traits, and/or measures relating to prejudice, stigma and intergroup anxiety. For both parts A and B, studies were excluded if they focused on non-fictional portrayals of autism, such as reality shows and news media reports.

### Identification of records

All results from the database searches were imported into Covidence software for managing and streamlining the systematic review.

### Record screening

**Figure 1.**
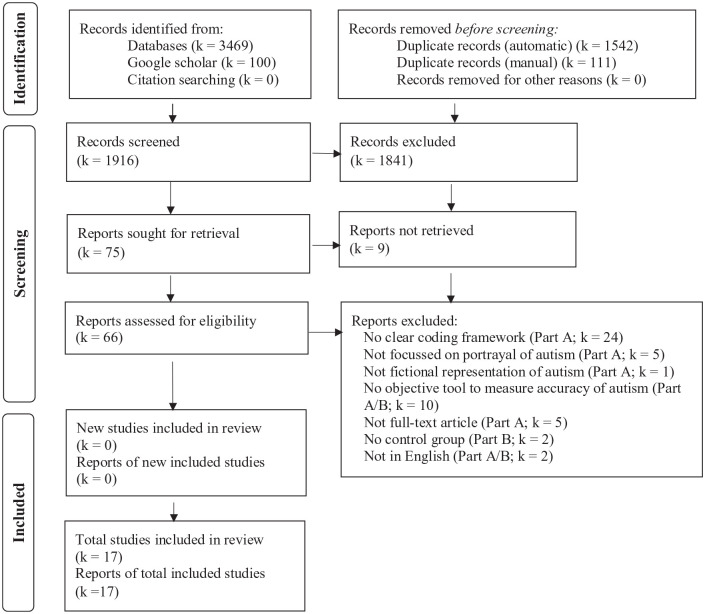
Adapted PRISMA 2020 flow diagram for the systematic review. PRISMA: Preferred Reporting Items for Systematic Review and Meta-Analysis.

Two researchers (CG, SM) independently reviewed titles and abstracts of the 1916 records against the inclusion and exclusion criteria. In cases of disagreement, a third researcher (SJ) reviewed the articles. The same process was followed for full-text screening, with an inter-rater reliability of 90% (see Figure 1 for PRISMA 2020 flow diagram).

### Data extraction and analysis

Preliminary data were screened and extracted from a random sample of six full-text articles (40%) by one of two reviewers. Based on this pilot, the data extraction plan was adjusted, with the primary change being combining results for the accuracy and authenticity of portrayals due to difficulty distinguishing between the two definitions. Data were extracted from each eligible study using a standardised Excel form.

### Quality appraisal

An adapted version of the Total Quality Framework (TQF) (Roller & Lavrakas, 2015) was used to assess the quality of the studies in Part A. This framework was selected as it relates specifically to qualitative content analysis where importance is placed on context and meaning. The TQF approach considers the entire research process and has four categories: ‘Credibility’ which considers the completeness and accuracy of the data collection phase, ‘Analysability’ which considers the procedures that were used to conduct a thorough and transparent interpretation of the data, ‘Transparency’ which focuses on how comprehensively the details of the study findings are discussed, and ‘Usefulness’ which concentrates on the extent to which the findings can be used to improve outcomes (Roller, 2019). An additional item, regarding whether the experience of coder(s) was stated, was added to the TQF for further quality appraisal; see Supplementary File 2.

An adapted version of the Critical Appraisal Skills Programme Randomised Controlled Trial Checklist (Critical Appraisal Skills Programme, 2018) was used to assess the quality of the studies in Part B. This checklist assesses the validity, methodological soundness and clarity of the results of randomised controlled trials; see Supplementary File 3. The section on ‘applicability’ was not included as the focus of the review was on assessing the state of the literature rather than assessing whether the results will help locally.

### Community involvement statement

There was no community involvement in this literature review study. However, the first author is an autistic autism researcher, and the article was reviewed by three members of the research centre’s autistic peer review panel prior to submission.

## Results

A total of 17 articles met the inclusion criteria and were included in the review: 14 for Part A and 3 for Part B (see Figure 1).

### How accurate and/or authentic are fictional media portrayals of autism? (Study Part A)

#### Description of studies

The majority (*n* = 11) of the 14 studies were peer-reviewed journal articles, while 3 (Ficarrotta, 2018; O’Neal, 2013; Wolff, 2018) were dissertations. In total, 32 movies, 28 television shows, 17 novels, 15 picture books and 7 online fanfiction articles were reviewed across the 14 studies, representing 106 autistic fictional characters across the 99 media depictions. Each study reviewed between 1 and 26 different types of media, with a mean of 10. The majority of depictions were reviewed in one to three studies, with the exception of Parenthood (TV show) and My name is Khan (film) which featured in four studies (see Supplementary File 4). One study (Black et al., 2019) did not state the year that the media included in the studies were released; for the remaining 13, the media reviewed covered a 30-year period from 1988 to 2017.

#### Canon versus coded

**Figure 2.**
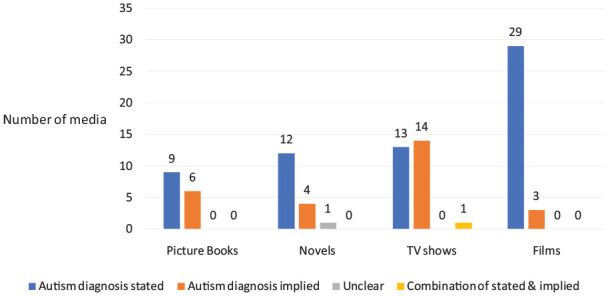
Canon versus coded.

Five of the studies (Black et al., 2019; Ejaz, 2020; Ficarrotta, 2018; Holton, 2013; Poe & Moseley, 2016) only included media where the character was canonically autistic (i.e. formally/officially identified as such), while one study (Maich et al., 2020) only included media where the character was coded as autistic or referred to as such in popular media and discourse (but not described as autistic in the show or its promotional material). Six studies (Garner et al., 2015; Kelley et al., 2018; Maich, 2014; Nordahl-Hansen, Tøndevold, & Fletcher-Watson, 2018; O’Neal, 2013; Wolff, 2018) included a combination of the above two categories, while this detail was not stated for two of the studies (Cardon & Kelley, 2016; Kelley et al., 2015). Comparing media types, the character’s autism diagnosis was explicitly stated in all fanfiction literature (*n* = 7), in most films and novels (*n* = 25 and *n* = 10, respectively), and about half of the TV shows and picture books (*n* = 13 and *n* = 6, respectively); see Figure 2. One TV show (House) had two autistic characters: one character’s diagnosis was explicitly stated and one character’s diagnosis was implied.

#### Coding framework used

Five of the studies used diagnostic tools as the coding framework: the Childhood Autism Rating Scale (CARS2-ST; (Ficarrotta, 2018; Garner et al., 2015) and/or the DSM-5 (Ficarrotta, 2018; Kelley et al., 2018; Kelley et al., 2015; Nordahl-Hansen, Tøndevold, & Fletcher-Watson, 2018). Ten studies utilised purpose-built coding frameworks.

Garner et al. (2015) reviewed 15 films from the period 1988 to 2010 and reported that 13 of the films scored in the *severe symptoms of autism* category on the CARS2-ST. The two films which were placed in the *minimal-to-no* and *mild-to-moderate* symptom categories, respectively, were *Bless the Child* (2000) and *Guarding Eddy* (2005); however, Bless the Child was difficult to rate due to a dramatic shift in idiosyncrasies which contributed to an unrealistic representation of autism.

Kelley et al. (2015) reviewed 15 fictional picture books published between 1996 and 2012, and Kelley et al. (2018) reviewed nine award winning novels published between 2004 and 2014, assessing characters against the DSM-5 criteria. Both studies found an overrepresentation of repetitive/restricted behaviours, such as stereotyped or repetitive motor movements (62% of characteristics in picture books and 72% in novels), and an underrepresentation of social communication deficits (38% and 28%, respectively). Older books were more likely to focus on restrictive and repetitive patterns of behaviour, whereas later publications tended to depict a variety of characteristics.

Nordahl-Hansen, Tøndevold and Fletcher-Watson (2018) reviewed 22 films and four TV series and found that characters typically demonstrated a very high match to DSM-5 criteria, with seven of the characters scoring the maximum possible on the total symptom scale. Savant-like skills were reported in 12 of the 26 characters, which is higher than actual prevalence. Ficarrotta (2018) used both measures to assess the character of Julia from Sesame Street, categorising her in the *mild-to-moderate level of behaviours related to autism* category of the CAR2-ST, and noting that she met the diagnostic criteria as she displayed examples of all three social communication and social interaction deficits and two of the four restrictive, repetitive behaviours.

**Figure 3.**
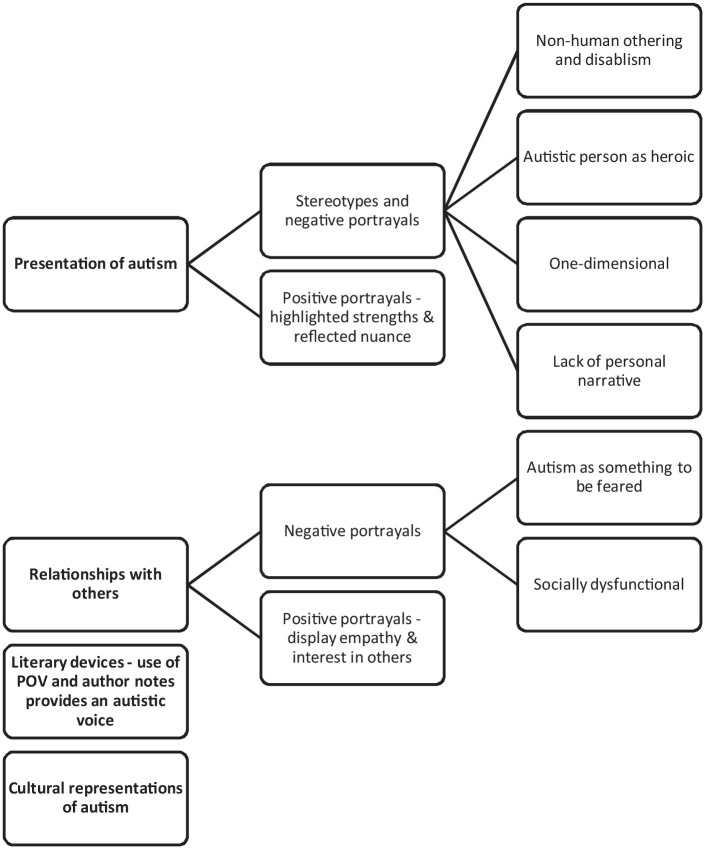
Representation of autism across the purpose-built frameworks.

Ten studies utilised purpose-built coding frameworks. The content coded within these studies could be grouped into four areas: how autism is presented, the relationship between the autistic character and other characters, literary devices used and cultural constructs; see Figure 3.

#### How autism is presented

Within presentation of autism, several stereotypical and negative portrayals were discussed. First, the presence of (non-human) othering and disablism (Ejaz, 2020; Holton, 2013; Maich et al., 2020; O’Neal, 2013), such as the autistic character in The Boy on the Bridge being referred to as ‘robotic’ and ‘the robot’ by his crewmates (Maich et al., 2020), and the use of terms like ‘crazy’, ‘retarded’, ‘handicapped’ and ‘stupid’ in Rain Man and I am Sam (Ejaz, 2020). Second, the presentation of autistic characters as having supernatural abilities (Holton, 2013; Maich, 2014; Maich et al., 2020; Poe & Moseley, 2016), with an overemphasis on characteristics such as the ‘savant’, ‘hero’ and ‘eccentric genius’. Third, a one-dimensional view of autism (Ejaz, 2020; Poe & Moseley, 2016; Wolff, 2018) that perpetuated stereotypes such as the ‘straight white male’ and overrepresentation of sensory issues as seen in examples such as Atypical (Wolff, 2018). Finally, autistic presentations had a lack of personal narrative (Holton, 2013; Poe & Moseley, 2016), with the autistic characters robbed of ‘agency’ and ‘independence’ (Poe & Moseley, 2016).

Positive portrayals of autism were those that highlighted strengths and reflected nuance. This included depicting characters who experienced discomfort with lying and ‘relentlessly pursu[ed] truth’ (O’Neal, 2013), and who were able to ‘grappl[e] with dilemmas’ they encountered in their day-to-day lives (Maich, 2014). Characters were also shown to have ‘intersect[ed] with other forms of difference’, such as dual diagnosis and other personal characteristics, which served to challenge the stereotypes of autistic persons and to demonstrate the uniqueness of each character (Black et al., 2019). Some characters further countered the myth that autistic people are not ‘attracted to others’ and highlighted their capacity to show empathy (Wolff, 2018). Importantly, positive portrayals used labelling and characteristics to educate and inform rather than to brand and expose (Ficarrotta, 2018).

#### Relationships between autistic character and other characters

In relation to the portrayal of the autistic character’s relationship with others, two negative aspects surfaced. First, the depiction of autism as something to be feared and as a burden (Holton, 2013; O’Neal, 2013; Poe & Moseley, 2016), particularly for the parents raising the autistic person, such as Max in Parenthood (Holton, 2013). Second was the representation of autistic characters as ‘socially dysfunctional’ (Poe & Moseley, 2016) and unable to form interpersonal relationships (Holton, 2013; Poe & Moseley, 2016), as seen in Fringe and Grey’s Anatomy (Poe & Moseley, 2016). In contrast, positive relational portrayals were those that highlighted the autistic character’s ability to display empathy and interest in others, including romantic interest (O’Neal, 2013; Wolff, 2018), such as Sam in Atypical (Wolff, 2018).

#### Literary devices and cultural contexts

The primary literary device discussed across two studies was the use of point-of-view and author notes to give voice to autistic perspectives (Black et al., 2019; Wolff, 2018). For example, Shaun’s internal monologue was shown through graphics in The Good Doctor (Black et al., 2019). Other literary devices used in positive ways were plot devices to educate the audience about prejudice and mistreatment experienced by autistic people (Black et al., 2019) and the strengths of different modalities, such as chapter books for providing more in-depth explanations of the characters and their autism (Cardon & Kelley, 2016). Ficarrotta (2018) highlighted Sesame Street’s reliance upon ‘episodic’ frames which focused on the autistic character’s circumstances rather than ‘thematic’ frames which focused on autism at a societal level. Finally, Ejaz (2020) compared cultural representations of autism across international film remakes finding a higher percentage of scenes including characters with disability in the American versions, and a higher percentage of scenes in which characters were depicted in a romantic setting or working and able to perform their duties in Indian versions.

#### Quality appraisal

All studies included in Part A identified a target population, relevant constructs and had a clear coding framework. Across the studies, there were limitations in the transparency of the reporting, with no studies reporting the coders’ reflections on the coding form. Only one study (Black et al., 2019) reported techniques used to identify categories and themes, and the verification approaches used to support or refute preliminary interpretations. The absence of this detail limits the extent to which the outcomes of the research can be critically evaluated and the replicability of the studies (Roller, 2019). See Supplementary File 2 for further detail.

### Impact of viewing fictional media portrayals of autism on knowledge about autism and attitudes towards autistic individuals? (Study Part B)

#### Description of studies

Two of the three publications included in Part B were dissertations, and the third was a peer-reviewed journal article (Stern & Barnes, 2019). Five studies were represented across the three publications; Stern (2020) included three studies. The sample size across the five studies ranged from 121–157, the mean age was 20.0 years and mean percentage of females was 69.9. All five studies used an experimental design with randomisation. One of the studies (Zhong, 2020) was conducted in an online context, while the other four were conducted in a university context; all studies were conducted in the United States.

Three of the studies (Stern, 2020) used excerpts from novels as the intervention stimulus, while two studies used excerpts from television series as the intervention stimulus. For each study, the intervention and control stimulus were a comparable length. See Table 2 for further detail on the stimuli used.

**Table 2.** Description of stimuli used in Part B studies.

Citation	Study	Exposure quantity (intervention & control)	Intervention			Control group stimulus
			Media type	Media title	Stimulus focus	
Stern, 2019		28 min	TV series	The Good Doctor	Pilot episode; focus on autistic savant protagonist; 16 min spent discussing/illustrating autism; one aspect of *DSM*-5 explicitly discussed, illustrative examples of all *DSM*-5 criteria	Lecture on autism; delivered by psychology professor and practicing clinical psychologist; 28 min spent discussing/ illustrating autism; all *DSM*-5 criteria explicitly discussed, illustrative examples of five categories
Stern, 2020	1	2200–2300 words	Novel	The Curious Incident of the Dog in the Night-Time	Excerpt from the novel; first-person perspective of the autistic character; 2/5 *DSM*-5 criteria covered, all 7 social, behavioural or cognitive hallmarks of autism covered	Excerpt from textbook chapter on autism; included general description of autism, DSM-V criteria and aetiology; 4/5 *DSM*-5 criteria covered, all 7 social, behavioural or cognitive hallmarks of autism covered
	2	2100–2400 words	Novels	The Curious Incident of the Dog in the Night-Time (mystery) and The Rosie Project (romance)	Randomised to excerpt from one of two novels; both first-person perspective of autistic character; The Rosie Project illustrated 3/5 DSM-5 criteria	Randomised to textbook excerpt from study 1 or a second textbook chapter; both cover same content; second textbook explicitly stated 4/5 DSM-5 criteria
	3	2565–2592 words	Novel	The Rosie Project (adapted version)	Modified version of the Rosie Project excerpt used in study 2; contained answers to 25 knowledge assessment items	Modified version of the textbook excerpt used in study 1; contained answers to 25 knowledge assessment items
Zhong, 2020		15:34 min	TV series	Atypical	Formed using two clips from Season 2 of Atypical; focus on positive outcomes between the autistic character and his friends	TED Talk video on Autism; no interaction (intergroup contact) between autistic and non-autistic individuals present

#### Measures used

Stern and Barnes (2019) and study 1 in Stern (2020) used the 30-item behavioural checklist developed by White et al. (2019) to assess changes in knowledge. Study 2 in Stern (2020) used a 50-item test of knowledge in four domains: diagnosis/symptoms, aetiology, treatment and awareness of stigma (Harrison et al., 2017). Study 3 in Stern (2020) used a 24-item modified version of the Harrison et al. (2017) knowledge test. Knowledge changes were not measured in Zhong (2020).

Changes in attitude were assessed in Stern and Barnes (2019) and study 1 in Stern (2020) using a purpose-built measure that contained 20 positive and 20 negative traits. Study 2 in Stern (2020) used a 6-item social distancing scale on comfortability associating with an autistic person (Gillespie-Lynch et al., 2015). Study 3 in Stern (2020) used the aforementioned purpose-built tool and social distancing scale. Zhong (2020) used three measures: 6-item adapted scale on prejudice against autistic individuals (Akrami et al., 2006), 5-item adapted scale on willingness for future contact with autistic individuals (Esses & Dovidio, 2002), and 6 items adapted from an intergroup anxiety scale that measures comfortability interacting with autistic people (Stephan & Stephan, 1985).

#### Results

There were no significant differences in number of behaviours *correctly* selected as being related to autism across the four studies. In Stern and Barnes (2019) and study 1 of Stern (2020), participants in the fiction condition *incorrectly* selected significantly fewer behaviours as associated with autism (i.e. those not typically associated with autism) than those in the lecture condition. For study 2 of Stern (2020), participants in the fiction condition were nearly twice as likely to select ‘I don’t know’ rather than identify an item as correct or incorrect. There were no significant changes in knowledge in the third study reported in Stern (2020).

In Stern and Barnes (2019) and studies 1 and 3 of Stern (2020), participants in the fiction conditions were significantly more likely to attribute positive traits to an autistic person than those in the control conditions, with medium effect sizes ranging from 0.57 to 0.73 (see Supplementary File 5). In Stern and Barnes (2019), participants in the fiction condition attributed significantly *fewer* negative traits to an autistic person than those in the control condition. In study 3 of Stern (2020), participants in the control condition showed a significant *increase* in number of negative traits attributed and a *decrease* in number of positive traits attributed post reading. Participants in the fiction condition showed significantly less desire for social distance after reading, while there was no change for the control condition. There were no significant changes in attitudes for study 2 of Stern (2020) or (Zhong, 2020).

#### Quality appraisal

The five studies reported across the three publications were comparable in terms of quality, with all studies reporting relevant elements of the study design and comprehensively reporting the results. None of the studies stated using blinding and only one study reported a comparison of the groups pre-intervention (Stern & Barnes, 2019). Zhong (2020) was the only study to report confidence intervals, which demonstrate what effects are likely to exist in the population and are thus recommended to be reported alongside p-values (Capraro, 2004). See Supplementary File 3 for further detail.

## Discussion

### Nature of autism representation in fictional media

The representation of autistic people in fictional media remains problematic. The studies included in this review found that portrayals of autism in film tend to focus on individuals with high support needs (e.g. full-time care and support), thus disregarding the varying levels of support that may be required by different individuals on the spectrum and the fluctuations in the amount of support required within the life span of an autistic person. Studies also contained explicit or implicit disablism, and consistent with findings from previous reviews, television series tended to focus on stereotypical portrayals of the autistic savant (Dean & Nordahl-Hansen, 2021; Fontes & Pino-Juste, 2021). Across all mediums, there was an overrepresentation of repetitive/restricted behaviours and an underrepresentation of social communication deficits; although, somewhat encouragingly, recent novels, picture books and television series were more likely to provide a nuanced representation of autism (Kelley et al., 2018; Kelley et al., 2015; Wolff, 2018).

While previous reviews focused on examining the demographic attributes of autistic characters (Dean & Nordahl-Hansen, 2021) and how autism is portrayed (Fontes & Pino-Juste, 2021), this review extended the examination of autism across a range of fictional media by additionally exploring the nature of relationships between autistic people and others, and how literary devices and culture context were used to provide autistic perspectives. Autistic characters in fiction continued to be presented as burdens or saviours, there to drive the plot or to evoke sympathy for their neurotypical families and friends, rather than as holistic individuals with agency. Even where the studies identified (what they considered to be) positive portrayals, they noted missed opportunities to educate viewers/readers, such as the sole focus on the characteristics of the autistic individual rather than on the social and environmental barriers to their inclusion and participation in society. The use of internal monologues in more recent television series (e.g. The Good Doctor, Atypical) was noted as being useful in providing further insight into the point-of-view of autistic characters.

While there is no ‘perfect’ or ‘ideal’ portrayal of autism–given that each portrayal can only represent one autistic individual and that every autistic person is different–there are clear areas for improvement. For example, diversity in demographics (not all autistic people are white heterosexual males), representation of the autistic perspective, and holistic portrayals of autistic people that are nuanced and display the strengths of the autistic person (Barrio et al., 2021).

### Impact of autism representation in fictional media

The four studies that measured autism knowledge pre- and post-exposure found few or no improvements in knowledge after viewing the fictional portrayal. One found no change, two found no change in correct identification of behaviours but a decrease in incorrect identification of behaviours, and one found an increase in ‘don’t know’ responses. In interpreting these results it is important to acknowledge the limitations in both the experimental exposure and the measurement instrument. All four of the studies that measured knowledge utilised a short exposure, a 28 min episode of a television series (Stern & Barnes, 2019) or a 2100–2600 word excerpt, or modified excerpt, from a novel (Stern, 2020). Stern and Barnes (2019) and studies 1 and 2 of Stern (2020) had limited coverage of the issues included in the knowledge measure.

Two of the studies used some or all of the items from White et al. (2019), a stereotype-laden and deficit-focused instrument that, for example, positions as *correct* descriptors of autistic college students ‘frequent interruptions of professor’ and ‘odd/awkward behaviour’. This same instrument positions as an *incorrect* descriptor of autistic college students ‘trouble learning’ based on a very ableist assumption that this is ‘not typical for students who have been accepted and are enrolled in higher education’ (White et al., 2019, p. 2701). Despite numerous strengths, self-reported experiences of autistic students in higher education have indicated a number of support needs related to information processing speed, time management, group work, presentations, motivation to study, following lectures and asking questions (Anderson et al., 2017; MacLeod & Green, 2009; Van Hees et al., 2015). We would argue that an ‘increase’ in knowledge score as measured by White et al. (2019) would not necessarily reflect a positive change in the respondent’s knowledge of autism.

There was limited evidence of positive attitude change as a result of exposure to fictional media portrayals of autism. Of the four studies that included pre- and post-exposure attitudinal measures, only one reported a change in reported attitudes (an increase in the number of positive traits attributed). The study with post-only measures reported more positive attitudes in the experimental than the control group; however, given that one of the pre–post studies reported an increase in negative attitudes in the control (text book) condition, it is equally possible that these differences reflect a negative impact of the control condition rather than a positive impact of the experimental condition. It is also important to note that all five studies utilised a stereotypical portrayal of autism: an intelligent (or savant) white heterosexual male.

The discernible lack of improvement in attitude and knowledge of autism highlights the issues in the portrayal of autistic characters in fictional media thus far, which is most likely to impact those under the age of 40 who are more likely to consume fictional media (particularly television and film; Deloitte, 2021). This is especially an issue for younger consumers as exposure to problematic portrayals of autism may impact their early perceptions of autistic persons, which provides the foundation to the formation of negative attitudes that may persist throughout their life span (Favazza et al., 2017).

### Limitations

This review sought to understand the accuracy and authenticity of fictional media portrayals of autism (Part A), and the impact of viewing fictional media portrayals of autism on knowledge about autism and attitudes towards autistic individuals (Part B). The heterogeneity of the studies in Part A, for example, differences in the coding frameworks used, made it difficult to compare findings across studies and media depictions. However, a strength of the study was the inclusion of the broad range of fictional media that audiences are exposed to, allowing for comparisons across media types. The small number of studies that met the inclusion criteria for Part B meant that a meta-analysis was not possible. Findings should also be considered preliminary given the small number of studies included in Part B.

### Future research

It is evident that portrayals of autism in fictional media are increasing, and for many people, entertainment media is their primary source of information about autism. Part A of this study found that media portrayals remain problematic, but preliminary evidence indicated that this may be changing. Future research should explore changes in the accuracy, complexity and diversity of autism portrayals (e.g. whether there is an increase in the portrayal of female autistic characters) over time and across media, and how this compares to changes in the knowledge and acceptance of autism of the wider community.

It is still unclear whether, how and to what extent these fictional portrayals impact on people’s awareness, understanding and acceptance of autistic people. As such, it is difficult to develop principles to provide guidelines to how fictional representations of autism can be used to improve knowledge and acceptance of autism in the wider community. While experimental studies, such as those included in this review, allow for controlled comparisons between groups, they were limited in number and did not replicate the experience of natural exposure to fictional media. People typically do not watch one episode of a television series or read one section of a book; neither do they consume and process media input alone or under controlled conditions. Understanding the real impacts of exposure to media portrayals of autism needs a combination of experimental studies, such as those reviewed here, and field research with people who have consumed this media in a natural setting. Findings from such studies will be critical in guiding the formation of guidelines and/or principles to how fictional media can be used to improve knowledge and acceptance of autism across varying contexts (e.g. in educational institutions).

If we are to truly understand the impacts of fictional media representations on autism awareness and acceptance, we need first to develop measures that accurately and respectfully measure autistic people’s experiences of community knowledge and attitudes (Jones, 2022a). We found that several of the measures used in the studies in this review reflect ableist, deficit-based and outdated perceptions of autism.

There is a need to explore the differential effects of canon vs coded portrayals of autism as both were included in several of the studies reported in Part A. The majority of studies in Part B used canon portrayals (The Rosie Project, Atypical, The Good Doctor), although two included a coded portrayal that is commonly but incorrectly identified as canonical (Christopher’s diagnosis is *not* explicitly stated in The Curious Incident). Coded portrayals offer media producers more flexibility but may have less educational value; an accurate portrayal of autism will not increase autism knowledge if the viewer is not aware that is what is being portrayed. Concerns have also been raised by autistic people that coded portrayals permit the presentation of autistic characters as objects of humour or derision in a way that would not be permitted if the character was identified as autistic (Jones 2022b).

Finally, media effects are likely to be cumulative and to interact with other exposures, including whether the individual has the opportunity to utilise their ‘knowledge’ in their interactions with autistic people. Thus, the field could benefit from longitudinal studies that consider multiple exposures to different fictional media portrayals and other sources of information about autism. This could include the representation of autistic people on social media platforms and in non-fiction (e.g. autobiographies, documentaries, news articles).

## Supplemental Material

sj-docx-1-aut-10.1177_13623613231155770 – Supplemental material for Representation of autism in fictional media: A systematic review of media content and its impact on viewer knowledge and understanding of autism Click here for additional data file. Supplemental material, sj-docx-1-aut-10.1177_13623613231155770 for Representation of autism in fictional media: A systematic review of media content and its impact on viewer knowledge and understanding of autism by Sandra C Jones, Chloe S Gordon and Simone Mizzi in Autism

sj-docx-2-aut-10.1177_13623613231155770 – Supplemental material for Representation of autism in fictional media: A systematic review of media content and its impact on viewer knowledge and understanding of autism Click here for additional data file. Supplemental material, sj-docx-2-aut-10.1177_13623613231155770 for Representation of autism in fictional media: A systematic review of media content and its impact on viewer knowledge and understanding of autism by Sandra C Jones, Chloe S Gordon and Simone Mizzi in Autism

sj-docx-3-aut-10.1177_13623613231155770 – Supplemental material for Representation of autism in fictional media: A systematic review of media content and its impact on viewer knowledge and understanding of autism Click here for additional data file. Supplemental material, sj-docx-3-aut-10.1177_13623613231155770 for Representation of autism in fictional media: A systematic review of media content and its impact on viewer knowledge and understanding of autism by Sandra C Jones, Chloe S Gordon and Simone Mizzi in Autism

sj-docx-4-aut-10.1177_13623613231155770 – Supplemental material for Representation of autism in fictional media: A systematic review of media content and its impact on viewer knowledge and understanding of autism Click here for additional data file. Supplemental material, sj-docx-4-aut-10.1177_13623613231155770 for Representation of autism in fictional media: A systematic review of media content and its impact on viewer knowledge and understanding of autism by Sandra C Jones, Chloe S Gordon and Simone Mizzi in Autism

sj-docx-5-aut-10.1177_13623613231155770 – Supplemental material for Representation of autism in fictional media: A systematic review of media content and its impact on viewer knowledge and understanding of autism Click here for additional data file. Supplemental material, sj-docx-5-aut-10.1177_13623613231155770 for Representation of autism in fictional media: A systematic review of media content and its impact on viewer knowledge and understanding of autism by Sandra C Jones, Chloe S Gordon and Simone Mizzi in Autism
