# Supplementary material for: Representation of autism in fictional media: A systematic review of media content and its impact on viewer knowledge and understanding of autism
Source: Autism. 2023 Feb 19;27(8):2205–17. doi: 10.1177/13623613231155770 (PMC10576905; doi:10.1177/13623613231155770)
Supplement: sj-docx-1-aut-10.1177_13623613231155770 – Supplemental material for Representation of autism in fictional media: A systematic review of media content and its impact on viewer knowledge and understanding of autism [file sj-docx-1-aut-10.1177_13623613231155770.docx]

**Supplementary file 1 – full search strategy for each database**

**CINAHL**

| 1 | TI ( {media} OR tv OR television OR entertainment OR movie* OR film* OR fiction* OR "popular culture" OR drama ) OR AB ( {media} OR tv OR television OR entertainment OR movie* OR film* OR fiction* OR "popular culture" OR drama ) |
| --- | --- |
| 2 | (MH "Motion Pictures") OR (MH "Television") |
| 3 | (MM "Motion Pictures") OR (MM "Television") |
| 4 | TI ( ASD OR autis* OR asperger* ) OR AB ( ASD OR autis* OR asperger* ) |
| 5 | (MH "Autistic Disorder") |
| 6 | (MM "Autistic Disorder") |
| 7 | TI ( knowledge OR attitude* OR influenc* OR perception OR stigma OR understand* OR awareness OR belief OR portray* OR stereotyp* OR depict* OR represent* OR presentation* OR fram* ) OR AB ( knowledge OR attitude* OR influenc* OR perception OR stigma OR understand* OR awareness OR belief OR portray* OR stereotyp* OR depict* OR represent* OR presentation* OR fram* ) |
| 8 | S1 OR S2 OR S3 |
| 9 | S4 OR S5 OR S6 |
| 10 | S7 AND S8 AND S9 |

Limits: English language

Results: 173

**PSYCINFO**

| 1 | TI ( {media} OR tv OR television OR entertainment OR movie* OR film* OR fiction* OR "popular culture" OR drama ) OR AB ( {media} OR tv OR television OR entertainment OR movie* OR film* OR fiction* OR "popular culture" OR drama ) |
| --- | --- |
| 2 | DE "Television" OR DE "Popular Culture" OR DE "Films" |
| 3 | MM "Television" OR MM "Popular Culture" OR MM "Films" |
| 4 | TI ( ASD OR autis* OR asperger* ) OR AB ( ASD OR autis* OR asperger* ) |
| 5 | DE "Autism Spectrum Disorders" |
| 6 | MM "Autism Spectrum Disorders" |
| 7 | TI ( knowledge OR attitude* OR influenc* OR perception OR stigma OR understand* OR awareness OR belief OR portray* OR stereotyp* OR depict* OR represent* OR presentation* OR fram* ) OR AB ( knowledge OR attitude* OR influenc* OR perception OR stigma OR understand* OR awareness OR belief OR portray* OR stereotyp* OR depict* OR represent* OR presentation* OR fram* ) |
| 8 | S1 OR S2 OR S3 |
| 9 | S4 OR S5 OR S6 |
| 10 | S7 AND S8 AND S9 |

Limits: English language

Results: 424

**Medline**

| 1 | TI ( {media} OR tv OR television OR entertainment OR movie* OR film* OR fiction* OR "popular culture" OR drama ) OR AB ( {media} OR tv OR television OR entertainment OR movie* OR film* OR fiction* OR "popular culture" OR drama ) |
| --- | --- |
| 2 | (MH "Television") OR (MH "Popular Culture") OR (MH "Motion Pictures") |
| 3 | (MM "Motion Pictures") OR (MM "Television") OR (MM "Popular Culture") |
| 4 | TI ( ASD OR autis* OR asperger* ) OR AB ( ASD OR autis* OR asperger* ) |
| 5 | (MH "Autism Spectrum Disorder") OR (MH "Autistic Disorder") |
| 6 | (MM "Autism Spectrum Disorder") OR (MM "Autistic Disorder") |
| 7 | TI ( knowledge OR attitude* OR influenc* OR perception OR stigma OR understand* OR awareness OR belief OR portray* OR stereotyp* OR depict* OR represent* OR presentation* OR fram* ) OR AB ( knowledge OR attitude* OR influenc* OR perception OR stigma OR understand* OR awareness OR belief OR portray* OR stereotyp* OR depict* OR represent* OR presentation* OR fram* ) |
| 8 | S1 OR S2 OR S3 |
| 9 | S4 OR S5 OR S6 |
| 10 | S7 AND S8 AND S9 |

Limits: English language

Results: 289

**ERIC (EbscoHost)**

| 1 | TI ( {media} OR tv OR television OR entertainment OR movie* OR film* OR fiction* OR "popular culture" OR drama ) OR AB ( {media} OR tv OR television OR entertainment OR movie* OR film* OR fiction* OR "popular culture" OR drama ) |
| --- | --- |
| 2 | (DE "Television") OR (DE "Films") OR (DE "Popular Culture") |
| 3 | TI ( ASD OR autis* OR asperger* ) OR AB ( ASD OR autis* OR asperger* ) |
| 4 | (DE "Autism") OR (DE "Asperger Syndrome") |
| 5 | TI ( knowledge OR attitude* OR influenc* OR perception OR stigma OR understand* OR awareness OR belief OR portray* OR stereotyp* OR depict* OR represent* OR presentation* OR fram* ) OR AB ( knowledge OR attitude* OR influenc* OR perception OR stigma OR understand* OR awareness OR belief OR portray* OR stereotyp* OR depict* OR represent* OR presentation* OR fram* ) |
| 6 | S1 OR S2 |
| 7 | S3 OR S4 |
| 8 | S5 AND S6 AND S7 |

Limits: English language

Results: 129

**Web of Science Core Collection**

| 1 | TI=( {media} OR tv OR television OR entertainment OR movie* OR film* OR fiction* OR "popular culture" OR drama ) OR AB=( {media} OR tv OR television OR entertainment OR movie* OR film* OR fiction* OR "popular culture" OR drama ) |
| --- | --- |
| 2 | TI=(ASD OR autis* OR asperger*) or AB=(ASD OR autis* OR asperger*) |
| 3 | TI=(knowledge OR attitude* OR influenc* OR perception OR stigma OR understand* OR awareness OR belief OR portray* OR stereotyp* OR depict* OR represent* OR presentation* OR fram*) or AB=(knowledge OR attitude* OR influenc* OR perception OR stigma OR understand* OR awareness OR belief OR portray* OR stereotyp* OR depict* OR represent* OR presentation* OR fram*) |
| 4 | 1 AND 2 AND 3 |

Limits: English language

Results: 648

**Scopus**

| 1 | ( TITLE-ABS-KEY ( {media} OR tv OR television OR entertainment OR movie* OR film* OR fiction* OR "popular culture" OR drama ) AND TITLE-ABS-KEY ( asd OR autis* OR asperger* ) AND TITLE-ABS-KEY ( knowledge OR attitude* OR influenc* OR perception OR stigma OR understand* OR awareness OR belief OR portray* OR stereotyp* OR depict* OR represent* OR presentation* OR fram* ) ) |
| --- | --- |

Limits: English language

Results: 3464
