# Supplementary material for: Representation of autism in fictional media: A systematic review of media content and its impact on viewer knowledge and understanding of autism
Source: Autism. 2023 Feb 19;27(8):2205–17. doi: 10.1177/13623613231155770 (PMC10576905; doi:10.1177/13623613231155770)
Supplement: sj-docx-2-aut-10.1177_13623613231155770 – Supplemental material for Representation of autism in fictional media: A systematic review of media content and its impact on viewer knowledge and understanding of autism [file sj-docx-2-aut-10.1177_13623613231155770.docx]

**Supplementary File 2.** Quality appraisal of Part A studies using the Total Quality Framework (TQF)

|  | 1 | 2 | 3 | 4 | 5 | 6 | 7 | 8 | 9 | 10 | 11 | 12 | 13 | 14 |
| --- | --- | --- | --- | --- | --- | --- | --- | --- | --- | --- | --- | --- | --- | --- |
| Credibility: *Scope* | | | | | | | | | | | | | | |
| Target population defined | ✓ | ✓ | ✓ | ✓ | ✓ | ✓ | ✓ | ✓ | ✓ | ✓ | ✓ | ✓ | ✓ | ✓ |
| Sampling approach defined | ✓ | ✓ | ✓ | ✓ | N/A^a^ | 🗶 | N/A^a^ | ✓ | ✓ | N/A^a^ | 🗶 | 🗶 | 🗶 | ✓ |
| Techniques used to maximise inclusion of sample to mitigate potential bias | ✓ | ✓ | ✓ | ✓ | N/A^a^ | ✓ | N/A^a^ | ✓ | 🗶 | N/A^a^ | 🗶 | 🗶 | 🗶 | 🗶 |
| Identified unit of analysis | ✓ | 🗶 | 🗶 | ✓ | ✓ | 🗶 | 🗶 | 🗶 | 🗶 | ✓ | 🗶 | 🗶 | 🗶 | 🗶 |
| Credibility: *Data gathering* | | | | | | | | | | | | | | |
| Relevant constructs are stated | ✓ | 🗶 | ✓ | ✓ | ✓ | ✓ | ✓ | ✓ | ✓ | ✓ | ✓ | ✓ | ✓ | ✓ |
| Coding framework stated | ✓ | ✓ | ✓ | ✓ | ✓ | ✓ | ✓ | ✓ | ✓ | ✓ | ✓ | ✓ | ✓ | ✓ |
| Codebook/coding instrument is used and/or training on how to code data is provided | ✓ | 🗶 | ✓ | ✓ | ✓ | ✓ | ✓ | ✓ | ✓ | ✓ | ✓ | 🗶 | 🗶 | 🗶 |
| Experience of coder/s stated* | 🗶 | ✓ | ✓ | 🗶 | 🗶 | ✓ | 🗶 | Partial | ✓ | 🗶 | ✓ | 🗶 | 🗶 | 🗶 |
| Consensus used to resolve coding conflicts | N/A^b^ | ✓ | N/A^b^ | ✓ | ✓ | N/A^b^ | N/A^b^ | ✓ | ✓ | 🗶 | N/A^b^ | N/A^b^ | 🗶 | N/A^b^ |
| Analysability*: Processing* | | | | | | | | | | | | | | |
| Categories/themes identified across codes | ✓ | ✓ | ✓ | 🗶 | ✓ | N/A^c^ | 🗶 | N/A^c^ | N/A^c^ | ✓ | N/A^c^ | ✓ | ✓ | ✓ |
| Analysability*: Verification* | | | | | | | | | | | | | | |
| Verification process undertaken (e.g., peer debriefing, discussing with colleagues) | ✓ | ✓ | 🗶 | Partial | ✓ | 🗶 | ✓ | ✓ | ✓ | 🗶 | 🗶 | 🗶 | 🗶 | 🗶 |
| Transparency/Reporting | | | | | | | | | | | | | | |
| Coders' reflections on the coding form, e.g., concerning problems they may have had in determining the appropriate code for particular content; | 🗶 | 🗶 | 🗶 | 🗶 | 🗶 | 🗶 | 🗶 | 🗶 | 🗶 | 🗶 | 🗶 | 🗶 | 🗶 | 🗶 |
| Techniques that were used to identify categories and themes, including the use of CAQDAS | 🗶 | ✓ | 🗶 | N/A^c^ | 🗶 | 🗶 | N/A^c^ | N/A^c^ | N/A^c^ | 🗶 | N/A^c^ | N/A^c^ | 🗶 | 🗶 |
| Specific verification approaches that were used to support or refute preliminary interpretations and the results of the verification process, i.e., the extent to which verification altered the researcher's preliminary findings | 🗶 | ✓ | 🗶 | 🗶 | 🗶 | 🗶 | 🗶 | 🗶 | 🗶 | 🗶 | 🗶 | 🗶 | 🗶 | 🗶 |
| Usefulness | | | | | | | | | | | | | | |
| Credibility issues discussed e.g., content sampling, unit of analysis | 🗶 | 🗶 | ✓ | ✓ | ✓ | ✓ | 🗶 | ✓ | ✓ | 🗶 | ✓ | 🗶 | ✓ | ✓ |
| Interpretations provided | ✓ | ✓ | ✓ | ✓ | ✓ | ✓ | ✓ | ✓ | ✓ | ✓ | ✓ | ✓ | ✓ | ✓ |
| Implications stated | ✓ | ✓ | ✓ | ✓ | ✓ | ✓ | ✓ | ✓ | ✓ | ✓ | ✓ | ✓ | ✓ | ✓ |
| *Note.* 1 = Belcher (2014); 2 = Black (2019); 3 = Cardon (2016); 4 = Ejaz (2020); 5 = Ficarrotta (2018); 6 = Garner (2015); 7 = Holton (2013); 8 = Kelley (2015); 9 = Kelley (2018); 10 = Maich (2020); 11 = Nordahl-Hansen (2018); 12 = O'Neal (2013); 13 = Poe (2016); 14 = Wolff (2018); ✓ = Yes; 🗶 = No; N/A = not applicable; * = additional item added to the TQF; N/A^a^ = there was no sample selection process as all media sources from the defined population were included; N/A^b^ = only one coder was involved; N/A^c^  = themes were not identified as they were pre-determined by the coding framework e.g., the DSM-IV diagnostic criteria; | | | | | | | | | | | | | | |
