# Supplementary material for: Representation of autism in fictional media: A systematic review of media content and its impact on viewer knowledge and understanding of autism
Source: Autism. 2023 Feb 19;27(8):2205–17. doi: 10.1177/13623613231155770 (PMC10576905; doi:10.1177/13623613231155770)
Supplement: sj-docx-3-aut-10.1177_13623613231155770 – Supplemental material for Representation of autism in fictional media: A systematic review of media content and its impact on viewer knowledge and understanding of autism [file sj-docx-3-aut-10.1177_13623613231155770.docx]

**Supplementary File 3.** Quality appraisal of Part B studies using the CASP RCT Checklist

|  | Stern (2019) | Zhong (2020) | Stern (2020) |
| --- | --- | --- | --- |
| Section A: Is the basic study design valid for a randomised controlled trial? | | | |
| Did the study address a clearly focused research question? | ✓ | ✓ | ✓ |
| Was the assignment of participants to interventions randomised? | ✓ | ✓ | ✓ |
| Were all participants who entered the study accounted for at its conclusion? | ✓ | ✓ | ✓ |
| Section B: Was the study methodologically sound? | | | |
| Were the participants ‘blind’ to intervention they were given? | 🗶 | 🗶 | 🗶 |
| Were the investigators ‘blind’ to the intervention they were giving to participants? | 🗶 | 🗶 | 🗶 |
| Were the people assessing/analysing outcome/s ‘blinded’? | 🗶 | 🗶 | 🗶 |
| Were the study groups similar at the start of the randomised controlled trial? | Partial | ? | ? |
| Apart from the experimental intervention, did each study group receive the same level of care (that is, were they treated equally)? | ✓ | ✓ | ✓ |
| Section C: What are the results? | | | |
| Were the effects of intervention reported comprehensively? | ✓ | ✓ | ✓ |
| Was the precision of the estimate of the intervention or treatment effect reported? | 🗶 | ✓ | 🗶 |
| *Note.* ✓ = Yes; 🗶 = No; N/A = not applicable; ? = unclear | | | |
