# Supplementary material for: Representation of autism in fictional media: A systematic review of media content and its impact on viewer knowledge and understanding of autism
Source: Autism. 2023 Feb 19;27(8):2205–17. doi: 10.1177/13623613231155770 (PMC10576905; doi:10.1177/13623613231155770)
Supplement: sj-docx-4-aut-10.1177_13623613231155770 – Supplemental material for Representation of autism in fictional media: A systematic review of media content and its impact on viewer knowledge and understanding of autism [file sj-docx-4-aut-10.1177_13623613231155770.docx]

**Supplementary File 4.** The number of studies that investigated each type of media
