# Supplementary material for: Representation of autism in fictional media: A systematic review of media content and its impact on viewer knowledge and understanding of autism
Source: Autism. 2023 Feb 19;27(8):2205–17. doi: 10.1177/13623613231155770 (PMC10576905; doi:10.1177/13623613231155770)
Supplement: sj-docx-5-aut-10.1177_13623613231155770 – Supplemental material for Representation of autism in fictional media: A systematic review of media content and its impact on viewer knowledge and understanding of autism [file sj-docx-5-aut-10.1177_13623613231155770.docx]

**Supplementary File 5.** Effect sizes for studies included in Part B

| Study | Outcome | Experimental group: mean (SD) | Control group: mean (SD) | p-value | Cohen’s d |
| --- | --- | --- | --- | --- | --- |
| Stern (2019) | knowledge (number of correct behaviours identified) | 8.03 (3.12) | 8.92 (2.72) | 0.56 | -0.30 |
| Stern (2019) | knowledge (number of incorrect behaviours identified) | 2.59 (2.12) | 4.69 (2.59) | <0.01 | -0.89 |
| Stern (2019) | attitudes (attribution of positive traits) | 7.35 (3.24) | 5.53 (3.12) | 0.01 | 0.57 |
| Stern (2019) | attitudes (attribution of negative traits) | 4.23 (2.53) | 5.64 (2.35) | <0.01 | -0.58 |
| Stern (2020) – Study 1 | knowledge (number of incorrect behaviours identified) | 4.23 (2.79) | 5.02 (3.19) | 0.04 | -0.26 |
| Stern (2020) – Study 1 | attitudes (attribution of positive traits) | 6.3 (3.15) | 3.95 (3.29) | <0.01 | 0.73 |
| Stern (2020) – Study 2 | knowledge (etiology) | 10.59 (2.14) | 11.82 (2.12) | <0.01 | -0.58 |
| Stern (2020) – Study 2 | knowledge (diagnostic/symptoms) | 11.45 (2.94) | 13.31 (1.80) | <0.01 | -0.76 |
| Stern (2020) – Study 2 | knowledge (stigma) | 6.04 (1.46) | 6.00 (1.29) | 0.91 | 0.03 |
| Stern (2020) – Study 2 | knowledge (treatment) | 9.03 (2.37) | 10.11 (2.08) | 0.04 | -0.48 |
| Stern (2020) – Study 2 | knowledge (number of correct behaviours identified) | 31.31 (5.91) | 35.43 (4.16) | <0.01 | -0.81 |
| Stern (2020) – Study 2 | knowledge (number of incorrect behaviours identified) | 4.01 (2.69) | 6.00 (2.74) | <0.01 | -0.73 |
| Stern (2020) – Study 2 | knowledge (number of 'I don't knows') | 12.90 (7.20) | 6.90 (4.70) | <0.01 | 0.99 |
| Stern (2020) – Study 2 | Desire for social distance from those with ASD | 1.46 (0.60) | 1.58 (0.56) | 0.93 | -0.21 |
| Stern (2020) - Study 3: | attitudes (attribution of positive traits) | 7.29 (2.54) | 5.31 (3.05) | No main effect of condition^ | 0.71 |
| Stern (2020) - Study 3: | attitudes (attribution of negative traits) | 4.00 (2.31) | 5.51 (2.63) | No main effect of condition^ | -0.61 |
| Stern (2020) - Study 3: | Desire for social distance from those with ASD | 1.57 (0.36) | 1.73 (0.72) | No main effect of condition^ | -0.28 |
| Stern (2020) - Study 3: | Knowledge of ASD (number of correct behaviours selected) | - | - | 0.73^~^ | - |
| Stern (2020) - Study 3: | Knowledge of ASD (selecting 'I don't know') | - | - | 0.46^~^ | - |
| Stern (2020) - Study 3: | Knowledge of ASD (number of incorrect behaviours selected) | - | - | 0.36^~^ | - |
| Zhong, 2020 | Prejudice towards autistic individuals | 3.05 (1.08) | 3.00 (1.17) | >0.05 | 0.33* |
| Zhong, 2020 | Willingness to engage in future contact with autistic individuals | 5.42 (1.16) | 5.22 (1.31) | >0.05 | 1.21* |
| *Note.* * = Cohen’s d extracted from main text; ^~^ = p value demonstrating main effect of condition in repeated measures ANOVA; ^ = as per repeated measures ANOVA. | | | | | |
